# Ozone therapy in musculoskeletal medicine: a comprehensive review

**DOI:** 10.1186/s40001-024-01976-4

**Published:** 2024-07-31

**Authors:** Madhan Jeyaraman, Naveen Jeyaraman, Swaminathan Ramasubramanian, Sangeetha Balaji, Arulkumar Nallakumarasamy, Bishnu Prasad Patro, Filippo Migliorini

**Affiliations:** 1 Department of Orthopaedics, ACS Medical College and Hospital, Dr. MGR Educational and Research Institute, Chennai, Tamil Nadu 600077 India; 2 Department of Orthopaedics, Government Medical College, Omandurar Government Estate, Chennai, Tamil Nadu 600002 India; 3 Department of Orthopaedics, Jawaharlal Institute of Postgraduate Medical Education and Research (JIPMER)–Karaikal, Puducherry, 605006 India; 4 Department of Orthopaedics, All India Institute of Medical Sciences, Bhubaneswar, Odisha 751019 India; 5 Department of Orthopaedic, Trauma, and Reconstructive Surgery, RWTH University Medical Centre, Pauwelsstraße 30, 52074 Aachen, Germany; 6 Department of Orthopaedic and Trauma Surgery, Academic Hospital of Bolzano (SABES-ASDAA), 39100 Bolzano, Italy; 7 Department of Life Sciences, Health, and Health Professions, Link Campus University, Rome, Italy

**Keywords:** Orthopaedics, Traumatology, Ozone, Musculoskeletal medicine, Treatment, Pain

## Abstract

Musculoskeletal disorders encompass a wide range of conditions that impact the bones, joints, muscles, and connective tissues within the body. Despite the ongoing debate on toxicity and administration, ozone demonstrated promise in managing several musculoskeletal disorders, modulating pain and inflammation. A literature search was conducted. The research design, methods, findings, and conclusions of the studies were then examined to evaluate the physiological effects, clinical application, controversies, and safety of the application of ozone in musculoskeletal medicine. Ozone application demonstrates considerable therapeutic applications in the management of musculoskeletal disorders, including fractures, osteoarthritis, and chronic pain syndromes. Despite these advantages, studies have raised concerns regarding its potential toxicity and emphasized the importance of adhering to stringent administration protocols to ensure safety. Additionally, heterogeneities in patient reactions and hazards from oxidizing agents were observed. Given its anti-inflammatory and analgesic qualities, ozone therapy holds potential in the management of several musculoskeletal disorders. Additional high-quality research with long follow-up is required to refine indications, efficacy and safety profile. Finally, for wider clinical acceptability and utilization, the development of international recommendations is essential.

## Introduction

Ozone therapy is an innovative modality which gained growing attention in medical science, especially in musculoskeletal medicine [1]. Administered as a mixture of oxygen and ozone gases, it stimulates physiological responses, particularly anti-inflammatory and analgesic effects [2, 3]. By doing so, ozone therapy is administered in various acute and chronic musculoskeletal disorders such as fractures [4–6], osteoarthritis (OA) [7–10], low back ache [11, 12], osteomyelitis [13–16], and chronic pain syndromes [17–19]. Beyond orthopaedic applications, ozone therapy has shown potential in treating conditions such as chronic fatigue syndrome (CFS) and myalgic encephalomyelitis (ME) using different modalities such as ozonated water, ozone oil, and ozone gas [20–24]. Tailored routes of administration, ranging from enteral ozonated oil [ingestion], enteral ozonated water [ingestion], parenteral oxygen/ozone gas mixture [intramuscular, subcutaneous, intratonsillar, intrathecal, intraperitoneal, periganglionic, intraforaminal, paravertebral, intra-articular, intradiscal, oral submucous, supralaminar, epidural, penile, and intravenous], parenteral ozonated water [intra-tumoral], systemic [minor autohemotherapy (MiAH), major autohemotherapy (MAH), extracorporeal blood oxygenation and ozonation (EBOO), intravenous, and rectal insufflation], topical ozonated water [wound wash, ozonized balneotherapy, mouth wash, irrigation (nasal, sinus, otological, vaginal, intrauterine, intestinal, and intravesical), and sauna therapy, topical oxygen/ozone gas mixture [insufflation (ontological, intrafistula, intrauterine, and intravesical) and hyperbaric bagging], topical ozonated oil [embrocation and inhalation], and topical ozonated saline solution [wound wash, mouth wash, irrigation (saline, ontological, vaginal, intrauterine, and intravesical)], highlight its versatility [18, 25–27]. In orthopaedic practice, intramuscular, subcutaneous, intrathecal, periganglionic, intraforaminal, paravertebral, intra-articular, intradiscal, oral submucous, supralaminar, epidural, intravenous routes are used. However, besides its therapeutic potential, concerns about ozone toxicity and the importance of adhering to safe administration protocols cannot be overlooked [21, 25, 26, 28]. Prolonged inhalation of tropospheric ozone has been linked to detrimental impacts on the respiratory system and vital organs, inducing chronic oxidative stress and inflammation across multiple organs [29]. However, in controlled therapeutic settings, ozone therapy has demonstrated calculated oxidative stress induction, which activates therapeutic benefits without resulting in acute or chronic toxicity [29]. Caution is warranted to ensure that ozone doses do not surpass the blood’s antioxidant capacity to prevent potential toxicity [30]. While ozone therapy has the potential to enhance erythrocyte characteristics, correct hypoxia in diseases, and increase ATP levels through glycolysis activation, precise application is essential to avoid toxicity concerns [30]. The existing evidence is divided between its clinical benefits and potential risks as an oxidizing agent, further nuanced by variability in patient responses [28]. Despite its environmental implications as a pollutant, when administered under controlled conditions, ozone therapeutic potential in musculoskeletal conditions, including OA and herniated discs, is documented [20, 25, 26]. Therefore, ozone therapy emerges as a potent yet intricate intervention, necessitating further in-depth scientific scrutiny.

Numerous studies have explored ozone therapy, yet discrepancies persist, yielding variable results. Existing literature on ozone therapy reveals research gaps: limited comparative studies between treatments, unclear safety and effectiveness in various musculoskeletal conditions and insufficient understanding of anti-inflammatory effects [3, 18, 31]. Reviews frequently concentrate on specialized areas, prompting the necessity for a comprehensive review to address lacunae. In light of these controversies, this review critically evaluates the current evidence on the utilization of ozone in musculoskeletal disorders.

## Effects of ozone

**Fig. 1:**
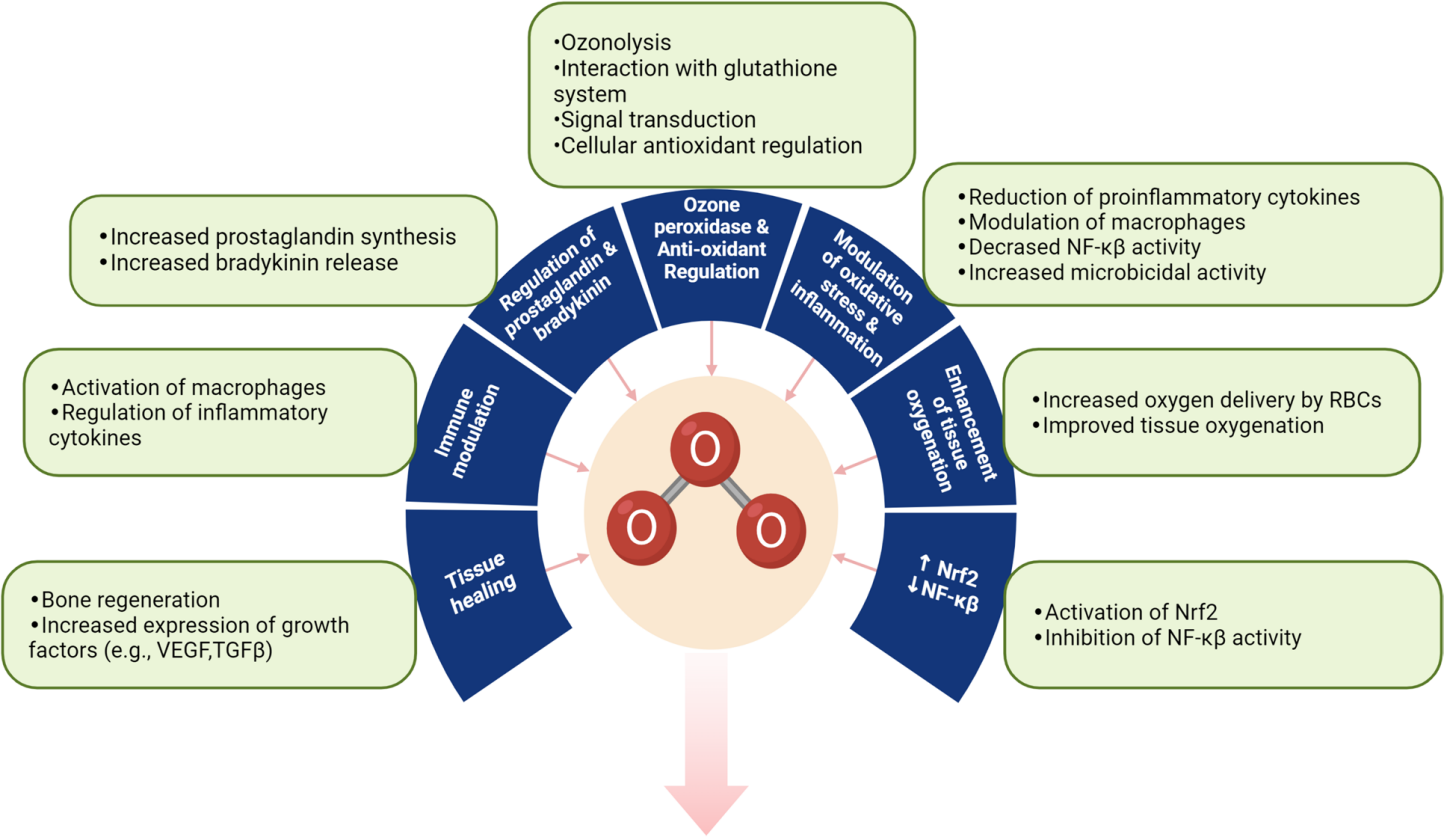
Physiological effects of ozone

Ozone therapy, with its multifaceted therapeutic capacities, has demonstrated notable physiological effects across various medical arenas. Its primary mechanism centres on enhancing tissue oxygenation, an indispensable component of cellular metabolism, by augmenting oxygen delivery to hypoxic tissues, thereby fostering optimal conditions for metabolic and repair processes [20, 26, 32]. For systemic administration of ozone, such as in major autohemotherapy (MAH), the concentration range should fall within 10–40 μg/mL, with 10–20 μg/mL of blood considered biologically relevant. It is advisable to administer a total ozone amount of 500–1000 μg per 50 mL of blood during MAH treatment. This induces the production of pharmacologically active hydroxy hydroperoxides, referred to as “ozone peroxides” [15, 25]. These compounds play a pivotal role in modulating endogenous antioxidant systems, particularly through interactions with the glutathione system [15]. A salient feature of ozone therapy is its proficiency in managing oxidative stress (Fig. 1), amplifying cellular antioxidant mechanisms while tempering the formation of reactive oxygen species (ROS) [3, 33]. Specifically, ozone engages with cysteine residues and glutathione, catalysing signal processes that support the bioregulation of enzymatic antioxidants [15]. This aptitude for oxidative stress modulation is important in conditions marked by persistent inflammation and immune system anomalies [3, 17]. Notably, ozone moderates inflammation by curtailing pro-inflammatory cytokines, such as interleukin 6 (IL-6), and simultaneously promotes anabolic activity, as evident by elevated insulin-like growth factor 1 (IGF-1) levels [33, 34]. In addition, ozone therapy exerts a regulatory effect on key transcription factors: it activates Nrf2, endorsing antioxidant and anti-inflammatory responses, while concurrently inhibiting NF-kB, integral in inflammation regulation [18, 33, 35, 36]. The therapy also modulates the synthesis of prostaglandins, impacts bradykinin release [26], and optimizes immune functions, particularly enhancing the activity of macrophages pivotal to immune reactions [3, 17]. From a tissue repair and angiogenesis standpoint, ozone therapy stimulates the expression of proteins such as collagen I, α-SMA, and TGF-β1, facilitating fibroblast activity [22]. It also upregulates growth factors, including vascular endothelial growth factor (VEGF) and transforming growth factor-beta (TGF-β), fostering enhanced tissue regeneration [23, 33]. Moreover, the activation of cellular signalling pathways like the PI3K/Akt/mTOR axis underscores its role in promoting the epithelial–mesenchymal transition (EMT) process [22, 37].

**Fig. 2:**
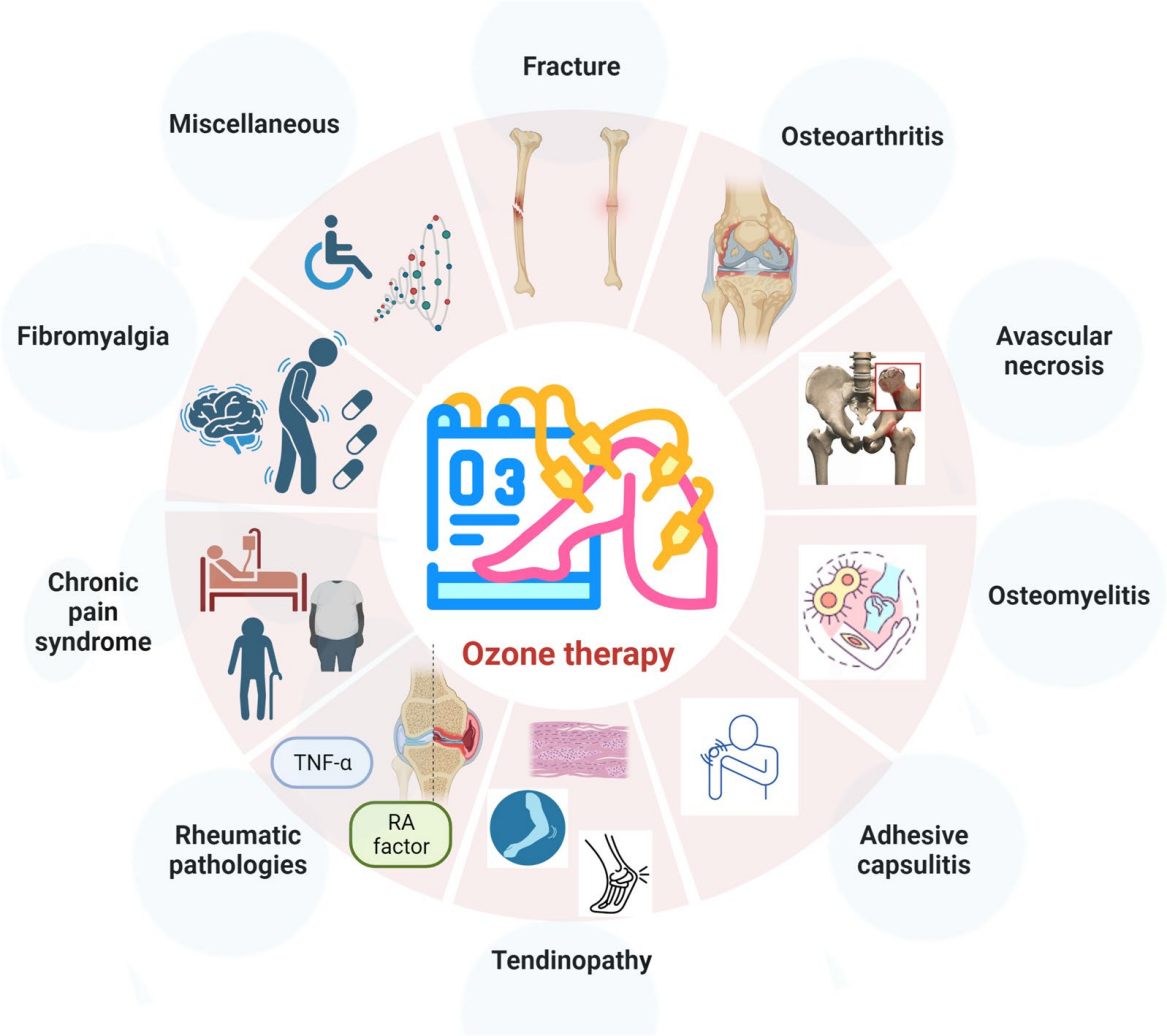
Applications of metamizole in musculoskeletal medicine

Clinically, ozone therapy has been effective in pain management, addressing conditions such as low back pain [18] and rotator cuff calcific tendinitis [38] (Fig. 2). Its influence extends to ameliorating microcirculatory disturbances seen in bone necrosis [1] and positively adjusting serum physiological markers, including total antioxidant capacity and lactate dehydrogenase [14], alongside inflammatory markers like the erythrocyte sedimentation rate [13]. The nuanced mechanisms of action underlying ozone therapy, ranging from improved oxygenation and oxidative stress modulation to anti-inflammatory effects, immune system enhancements, and tissue repair, validate its rising interest as a potential adjunctive treatment for several medical conditions. Its multifaceted physiological interactions render it an asset in contemporary clinical practice.

## Clinical applications of ozone in orthopaedics

### Fractures

Fracture non-union posed challenges in orthopaedic medicine, prompting the exploration of novel approaches [39]. In this context, ozone therapy garnered attention for its potential to enhance bone healing. Experimental studies using animal models, especially those with critical size defects, have demonstrated its efficacy. Duman et al. documented improved aspects of bone healing in a rat femoral fracture model with ozone therapy, noting enhanced union, bone formation, and bone marrow organization [40]. These findings align with those of Irban et al., who observed increased periosteal thickness and trabecular formation, thereby solidifying ozone therapy prospective role in bone regeneration [41]. These preclinical insights are bolstered by clinical evidence underscoring the therapy benefits. Shah et al. detailed a case wherein ozone therapy expedited wound healing and infection resolution over a tibial area, resulting in the patient regained ability to walk within 20 months [42]. Beyond fracture healing, ozone therapy has demonstrated effectiveness in pain management. Sconza et al. identified a comparable efficacy between ozone therapy and hyaluronic acid injections for knee OA pain alleviation and functional improvement [8]. Additionally, in comparison with low-level laser therapy, ozone therapy evidenced superior performance in promoting bone formation in rat calvarial defects [5]. Irban et al. conducted a comprehensive examination revealing ozone therapy profound impact on bone healing. Beyond the observed increase in periosteal thickness and trabecular areas, there was a marked elevation in vital growth factors and proteins, such as VEGF, β-catenin, and TGF-β, in the ozone-administered group versus the control [41]. Nevertheless, while the existing evidence is promising, it is crucial to address the current methodological limitations. Bennett et al. systematic review highlighted concerns regarding the quality of studies on the prevention or treatment of fracture non-union, pinpointing issues like lack of method standardization, inconsistent outcome measures, and inadequate reporting, which currently hampers the feasibility of meta-analyses [4].

### Osteoarthritis

The advent of ozone therapy modalities, specifically O_2_–O_3_ therapy and oxygen–ozone therapy (OOT), has significantly enriched the therapeutic spectrum for knee OA. These interventions, recognized for their ability to modulate oxidative stress and suppress pro-inflammatory cytokines, offer pain relief and functional enhancement in patients with knee OA [3, 43, 44]. Ozone therapy yields outcomes in pain, stiffness, and overall function comparable to the traditionally favoured hyaluronic acid injections [8]. Fernandez et al. observed its effectiveness through intra-articular injections for knee OA, resulting in symptomatic relief and improved physical function [33]. Comparative trials, such as those juxtaposing the efficacy of ozone prolotherapy and intra-articular hypertonic saline, found both modalities to be therapeutically analogous [45]. Periodic intra-articular ozone injections have been advocated for their consistent pain amelioration, joint function enhancement, and overall quality of life improvement, alongside a commendable safety profile [10]. These favourable findings merit a balanced perspective. A systematic review of randomized controlled trials (RCTs) pinpointed the generally subpar methodological quality in the prevailing research on this topic [4]. A previous review on OOT for knee OA reinforced this finding, suggesting that while short to medium-term results were encouraging, the included RCTs exhibited discernible methodological constraints [8]. When juxtaposing ozone therapy with hyaluronic acid injections, both modalities manifested significant advancements in pain, stiffness, and function across various time points, including 1, 3, and 6 months of follow-ups [46]. The anti-inflammatory and anabolic attributes of ozone therapy have garnered notable attention. Fernandez-Cuadros et al. documented reductions in inflammatory markers such as interleukin-6 (IL-6), C-reactive protein (CRP), erythrocyte sedimentation rate (ESR), and uric acid, coupled with pain alleviation and functional improvements spanning all grades of knee OA [33]. Farpour et al. findings echoed this, revealing that both ozone prolotherapy and intra-articular hypertonic saline injections offered parallel therapeutic benefits [45]. Moreover, a specific RCT highlighted the potential of weekly ozone injections over 8 weeks, emphasizing discernible pain reduction, improved joint functionality, and enhanced life quality [10]. While the prevailing literature accentuates the prospective benefits of ozone therapy in knee OA management, it simultaneously underscores methodological inadequacies in the current research landscape. To unequivocally establish ozone therapy standing as an efficacious knee OA treatment, there is an undeniable need for comprehensive, multicentre RCTs with extended follow-ups to corroborate its long-term benefits and reliability.

### Osteonecrosis of the femoral head

Ozone therapy has emerged as a promising alternative in treating osteonecrosis of the femoral head, especially for patients unresponsive to traditional therapeutic approaches. The therapy effectiveness is rooted in its capacity to promote neovascularization and immunoregulation, both pivotal for repairing hypoxic-ischaemic necrosis in the femoral head [1, 47]. Elucidating the molecular underpinnings of this treatment, An et al. highlighted the influence of differentially expressed genes, suggesting that individualized ozone therapy regimens, tailored to specific molecular markers, could be developed [1]. Complementary findings by An et al. showcased the augmented benefits of conjoining intra-articular O_2_–O_3_ hip injections with O_3_-AHT (autohemotherapy). Such a combination not only ameliorated enduring pain, but also aided in mitigating bone marrow oedema across diverse stages of osteonecrosis of the femoral head. Importantly, differential clinical outcomes between patients responsive and non-responsive to ozone therapy underscored the significance of immunoregulatory pathways, particularly spotlighting the impact of mitotically active lymphocytes in modulating treatment efficacy [1]. Branching out from osteonecrosis of the femoral head, the therapeutic scope of ozone therapy has been assessed in other conditions like avascular bisphosphonate-induced jaw osteonecrosis [48–51]. Agrillo et al. indicated that over half of the participants experienced full lesion healing and symptom abatement, with another 30% witnessing substantial lesion diminution and symptom relief. Even though lesions persisted relatively unchanged in 16% of the patients, they still reported some alleviation in symptoms, suggesting that ozone therapy might be an effective alternative to more invasive interventions, such as bone ablation surgery, particularly for bisphosphonate-induced related jaw osteonecrosis cases [51].

### Osteomyelitis

Ozone therapy potential as a treatment for osteomyelitis, a challenging bone infection often unyielding to conventional modalities, has garnered increasing attention in both clinical and preclinical domains. Yasheng et al. demonstrated the effectiveness of a treatment approach combining ozonated water lavage, physiological saline irrigation, and vacuum-sealed drainage for chronic osteomyelitis. This regimen yielded significant bacterial clearance and stimulated granulation tissue formation, attesting to ozone therapy potential as a reliable intervention [16]. Further, Shetty et al. highlighted its adaptability in specialized cases, showcasing its efficacy in neonatal mandibular osteomyelitis when combined with intravenous vancomycin antibiotics. The route of ozone administration involves the local application of ozonated water to the lesional site. The ozonated water is prepared by infusing three cycles of ozonated gas into 100 mL of saline over a 24-h p24 hours [52]. Animal studies bolster these clinical observations. Bilge et al., using a rat osteomyelitis model, noted improved histopathological parameters, suggesting ozone therapy has potent anti-inflammatory attributes [14]. Focusing on inflammatory markers, Nabi et al. reported the therapy positive impact on indices like the erythrocyte sedimentation rate, documenting a commendable 86.66% recovery in a patient cohort [13]. This line of exploration aligns with Shah et al. emphasis on ozone inherent disinfectant and antibacterial properties, positioning it as a beneficial option for extensive orthopaedic conditions, including osteomyelitis [42]. Furthermore, ozone therapy role extends to conditions such as bisphosphonate-related jaw osteonecrosis. Agrillo et al. evidenced complete healing in a significant patient proportion and substantial symptom relief in others [51]. An RCT delving into ozone therapy for chronic osteomyelitis showed the treatment group exhibiting marginally improved recovery rates and inflammatory indices, without notable adverse events, although the observed difference was not statistically significant [13].

### Adhesive capsulitis

Recent randomized controlled trials have sought to discern the optimal treatment strategy for adhesive capsulitis of the shoulder. Foula et al. compared the therapeutic merits of ultrasound-guided intra-articular injections, specifically contrasting ozone, steroid, and pulsed radiofrequency. The equipment utilized in the study employed platelet-rich fibrin (PRF) generators, which emit oscillating pulses spanning a frequency range of 420–500 kHz. These pulses have an amplitude of 45 V and a duration of 20 ms, followed by a silent period lasting 480 ms. Their results indicated the superior long-term efficacy of pulsed radiofrequency in symptom alleviation compared to its counterparts, ozone and steroid injections [53]. Another study echoed the benefits of these ultrasound-guided intra-articular injections in treating shoulder adhesive capsulitis. It documented marked improvements in pain, disability, range of motion (ROM), and inflammation across all intervention groups. Notably, visual analogue scale (VAS) scores during movement exhibited significant enhancements from the second week onward, persisting through the fourth and eighth weeks for all interventions. The steroid group, in particular, showed early improvements with VAS scores at rest enhancing from the initial week. ROM and the Shoulder Pain and Disability Index (SPADI) scores also reported progress from the second week. Moreover, consistent reductions in inflammatory markers, intercellular adhesion molecule-1 (ICAM-1), and high-sensitivity C-reactive protein (hs-CRP), were observed across the groups [53]. While both studies validate the efficacy of ultrasound-guided intra-articular injections in managing adhesive capsulitis symptoms, Foula et al. findings distinctly spotlight pulsed radiofrequency advantage for sustained symptom relief [53]. Concurrently, the subsequent study provides a more holistic perspective, underscoring the significant short-term benefits of both ozone and steroid injections, in addition to pulsed radiofrequency. Thus, pulsed radiofrequency stands out for its long-term therapeutic potential. Still, all modalities, including ozone and steroids, remain significant contenders in the short-term therapeutic landscape for adhesive capsulitis of the shoulder.

### Tendinopathies

Ozone therapy is progressively gaining prominence in managing various musculoskeletal conditions, especially tendinopathies [20, 38, 54–56]. Hidalgo-Tallon et al., spotlighted the therapy efficacy in treating subacromial tendinopathy, illustrating its advantage over mesotherapy and steroid injections [2]. Complementary research by Dong et al. [38] and Atar et al. [56] further substantiated these findings, suggesting ozone therapy potential in managing conditions like rotator cuff calcific tendinitis and chronic supraspinatus tendinopathy. These studies suggest that ozone injections are comparable to corticosteroid injections in alleviating symptoms. Further broadening the scope of ozone therapy, the modality matches corticosteroid injections in delivering significant relief from chronic plantar fasciitis [55]. This not only accentuates ozone therapy versatility, but also positions it as a potent alternative to established treatments. In the realm of sports medicine, the ozone therapy utility is becoming apparent. Hidalgo-Tallon et al. showcased ozone therapy benefits in managing hamstring injuries among professional athletes, noting pronounced pain reduction and functional improvements [57]. Seyam et al. highlighted positive outcomes from ultrasound-guided ozone therapy for partial supraspinatus tendon tears [20]. Furthermore, with an emphasis on compassionate care and treatment-resistant cases, Hidalgo-Tallon et al. proposed ozone therapy as an invaluable tool [2]. While ongoing research is geared towards elucidating ozone therapy comprehensive efficacy relative to corticosteroid injections in treating rotator cuff calcific tendinitis, definitive outcomes remain anticipated [38]. Additionally, animal studies such as the one by Gurger et al. spotlight the augmented effect of combining ozone therapy with platelet-rich plasma (PRP) on tendon-to-bone healing. This particular study revealed the duo potential in enhancing biomechanical strength, collagen fibre continuity, and alignment, thus hinting at the possibility of using ozonized PRP as a biological catalyst in tendon healing processes [58]. Lastly, Atar et al. randomized controlled trial buttressed the comparable therapeutic potency of ozone and corticosteroid injections for chronic supraspinatus tendinopathy in terms of pain relief, life quality, and functionality, though emphasizing the need for extended studies to gauge long-term outcomes [56].

### Rheumatic pathologies

Ozone therapy has emerged as a noteworthy intervention in the realm of rheumatic diseases, driven primarily by its capacities to modulate oxidative stress and attenuate pro-inflammatory cytokines [3]. Rheumatoid arthritis stands as a significant context where ozone therapy is increasingly being validated for methotrexate (MTX) efficacy for clinical response and improves cellular redox balance [59–61]. In animal models induced by Freund complete adjuvant and another experimental model, studies by Bozbas et al. and Zhao et al., respectively, observed marked attenuation of symptoms, histopathological signs of inflammation, and reductions in pro-inflammatory cytokines [62, 63]. Furthermore, Patel et al. postulate the potential dual application of ozone therapy, both as a primary treatment and synergistically alongside stem cell therapy or natural medicines [64]. Complementing its standalone utility, ozone therapy demonstrates promise as an adjunctive approach in conventional treatment protocols for rheumatoid arthritis. Fernandez et al. explored its potential combined with methotrexate (MTX), finding enhanced therapeutic outcomes of MTX in rheumatoid arthritis [61]. This combined approach not only mitigated disease activity, but also reinforced the antioxidant system, accentuating ozone therapeutic complementarity [61]. Beyond rheumatoid arthritis, the application spectrum of ozone therapy expands to other rheumatic conditions. Studies by Seyam et al. and Tartari et al. also spotlighted ozone therapy role in modulating oxidative damage in systemic sclerosis and its potential applicability in systemic autoimmune rheumatic diseases via cytokine profile adjustments [20, 60]. However, nuances exist in the broader application of ozone. Zhao et al. study pointed to a correlation between fine particulate matter (PM2.5) exposure and systemic autoimmune rheumatic diseases, yet did not firmly link ozone exposure with the onset of these diseases [63]. While another study affirmed ozone therapy benefits in reducing inflammation and arthritis severity in an animal model with rheumatoid arthritis, the alterations in oxidative stress markers remained statistically inconclusive [62].

### Chronic pain syndromes

Ozone therapy has solidified its position as an effective therapeutic modality in managing an array of chronic pain conditions. Particularly, intramuscular injections of the O_2_–O_3_ mixture have shown significant efficacy in alleviating cervicobrachial pain, with discernible reductions in VAS pain scores [65, 66]. Parallel to this, intramuscular paravertebral lumbar injections of the mixture have proven fruitful in addressing low back pain, as reflected by reductions in VAS scores and enhancements in the Oswestry Disability Index (ODI) scores [11]. This is further substantiated by studies by Andrade et al. and Biazzo et al., which emphasize the minimally invasive and effective nature of ozone therapy in lumbar pain management [11, 12]. A meta-analysis considering ozone therapy role in lumbar pain underscores its therapeutic potential but also signals the necessity for caution due to the high or uncertain risk of bias in some of the assessed trials [12]. Beyond specific pain syndromes, ozone therapy exhibits broader mechanisms, such as oxygenation, immune modulation, and anti-inflammatory action, and has been applied to other chronic conditions like ME/CFS and CFS [17, 67–69]. Furthermore, its efficacy has been observed to be comparable, if not superior, to standard treatments in Pain Units, especially when conventional therapies fall short [2]. The treatment adaptability was further highlighted in a case report detailing its successful application in an 11-year-old with Complex Regional Pain Syndrome and pseudo-seizures, marking a full remission post-intervention [17]. Delving into synergistic applications, Patel presented the intriguing prospect of combining ozone therapy with stem cell interventions, particularly for rheumatoid arthritis. However, this proposal is nascent and requires in-depth exploration for both safety and effectiveness [64]. While ozone therapy therapeutic potential is increasingly acknowledged, there exists a clear mandate for more robust scientific investigation. Tartari et al. emphasize the need for clarity in indications, protocol optimization, and pinpointing the patient demographics most likely to benefit [60]. Thus, despite the growing advocacy for ozone therapy as a versatile and efficient treatment modality in chronic pain management, its broad clinical application demands further empirical rigour.

### Fibromyalgia

Ozone therapy has emerged as a viable therapeutic option in addressing chronic conditions marked by fatigue and musculoskeletal pain, particularly myalgic encephalomyelitis (ME)/chronic fatigue syndrome (CFS) [67–70] and fibromyalgia [71–74]. In patients with ME/CFS, oxygen–ozone autohemotherapy (O_2_–O_3_-AHT) has been linked to significant clinical advantages. After O_2_–O_3_-AHT, approximately 43.5% of participants experienced a marked reduction in fatigue symptoms, with these benefits persisting for at least 3 months across diverse demographics [68]. The therapy impact on ME/CFS extends beyond symptom alleviation, showing potential in modulating metabolic pathways, oxidative stress, antioxidant systems, and immune and inflammatory responses [67]. Simultaneously, for fibromyalgia, a condition which shares many symptomatic parallels with ME/CFS, rectal insufflation ozone therapy has shown significant therapeutic potential [66]. Hidalgo-Tallon et al. observed substantial improvements in fibromyalgia patients’ physical symptoms, as reflected by decreased Fibromyalgia Impact Questionnaire scores within the initial 4 weeks of intervention [66]. In addition to physical improvements, the therapy also positively influenced psychological parameters, evident from reduced depression scores and enhanced Physical Summary Scores on the SF-12 questionnaire [66]. Subsequent research has affirmed these findings, with notable reductions in fatigue and psychological distress in fibromyalgia patients post-ozone therapy [25, 41]. While transient meteorism was occasionally reported, the side effects did not negate the overall beneficial outcomes of the therapy [66]. Furthermore, in a distinct study focusing on CFS, an impressive 70% of patients reported significant symptom alleviation, with the treatment presenting no major adverse effects [67]. Collectively, ozone therapy, whether through O_2_–O_3_-AHT or rectal insufflation, provides a robust therapeutic approach to the comprehensive challenges presented by ME/CFS and fibromyalgia. The therapy multifaceted mechanisms of action, coupled with its evident safety profile [67–69], advocate for its consideration, especially for patients who have found limited relief with other treatments [69].

### Miscellaneous

Ozone therapy has been identified as a versatile therapeutic modality, demonstrating efficacy across diverse medical conditions. For avascular bisphosphonate-related jaw osteonecrosis, approximately 54% of patients achieved complete lesion healing following an average treatment duration of 6.5 months, suggesting its potential in managing such conditions [51]. Furthermore, ozone therapy has demonstrated longer-lasting symptom relief for chronic plantar fasciitis compared to corticosteroids [55].

In orthopaedics, ozone therapy has been examined for its benefits in a range of conditions, from temporomandibular joint disorders to low back pain and carpal tunnel syndrome [8, 11, 15]. Its application has also been extended to chronic wound and ulcer management. Topical and injected ozone treatments have enhanced wound healing by reducing inflammation, facilitating rapid wound closure, and promoting angiogenesis and fibroblast proliferation, as evident from animal studies [22, 23]. Case studies and systematic reviews, such as those by Fitzpatrick et al. and Romary et al., highlight the efficacy of ozone therapy in promoting wound healing, especially in chronic wound scenarios [24, 75]. Particularly for diabetic foot ulcers, ozone therapy has been credited for reducing wound size and amputation rates [76–80].

The mechanistic attributes of ozone therapy in tissue repair have also been explored. Studies highlight its role in promoting fibroblast migration, stimulating epithelial–mesenchymal transition via the PI3K/Akt/mTOR pathway, and augmenting angiogenesis [22, 23]. A notable case study indicated the potential of combined ozone therapy in promoting the healing of extensive tibial wounds, enhancing the patient mobility [42]. In sports medicine, ozone therapy has proven valuable in managing injuries such as those in professional athletes with hamstring injuries, resulting in diminished pain, improved mobility, and enhanced perfusion [57, 77, 78]. Its safety and effectiveness are also corroborated by systematic reviews in pain medicine for conditions like knee OA [2]. The significant research findings and merits and de-merits of ozone therapy for various musculoskeletal conditions are jotted in Tables 1 and 2, respectively.

**Table 1:** Ozone interventions for various musculoskeletal conditions

Musculoskeletal condition	Significant findings
Fractures [5, 39–42]	• In animal models showed significant improvements in bone healing • Clinical case reports demonstrated effectiveness in treating extensive wounds
OA [33, 45, 46]	• Pain reduction and functional improvement in knee OA • Comparable efficacy to hyaluronic acid injections
Osteonecrosis of the femoral head [48–51]	• Potential in promoting neovascularization and immunoregulation • Treatment response determined by gene expression
Osteomyelitis [13, 14, 16, 42, 51, 52]	• Improvement in bacterial clearance and tissue healing • Effective in treating osteomyelitis in unique clinical presentations
Adhesive capsulitis [53]	• Pulsed radiofrequency was more effective than ozone therapy in long-term symptom relief
Tendinopathies [2, 20, 38, 54–58]	• Therapeutic effectiveness in various tendinopathies but with similar efficacy to corticosteroid injections
rheumatic pathologies [59–64]	• Potential in managing rheumatoid arthritis and systemic sclerosis with an enhanced efficacy when used in combination with methotrexate
Chronic pain syndromes [60, 65–69]	• Effectiveness in pain management across various conditions when comparable or superior efficacy to conventional treatments
Fibromyalgia [71–74]	• Effectiveness in managing symptoms and improving quality of life which shown to alleviate physical symptoms and reduce depression
Miscellaneous [2, 11, 15, 22–24, 42, 51, 55, 75–80]	• Potential in avascular bisphosphonate-related jaw osteonecrosis • Effective in musculoskeletal disorders, wound management, etc.

**Table 2:** Merits and de-merits of ozone therapy in musculoskeletal conditions

Merits of ozone therapy	De-merits of ozone therapy
• Promotes bone healing and enhances union, bone formation, and bone marrow organization in fractures • Effective in treating challenging cases of human fracture healing, including extensive wounds and infections • Comparable benefits in pain reduction and functional improvement to hyaluronic acid injections in knee OA • Superior efficacy in promoting bone formation compared to low-level laser therapy • Potential for personalized therapy based on differentially expressed genes in avascular necrosis • Effective in managing chronic osteomyelitis, promoting bacterial clearance and granulation tissue formation • Versatile in treating various tendinopathies, including subacromial tendinopathy, rotator cuff calcific tendinitis, and chronic plantar fasciitis • Potential applications in managing rheumatic diseases, including rheumatoid arthritis and systemic sclerosis • Effective in managing chronic pain syndromes, such as cervicobrachial pain, low back pain, complex regional pain syndrome, and fibromyalgia • Promising in treating conditions characterized by fatigue and musculoskeletal pain, such as ME/CFS and fibromyalgia • Effective in wound management, promoting wound closure, reducing wound size, and expediting healing, particularly in chronic wounds and ulcers	• Limited high-quality research: methodological limitations and small sample sizes • Lack of standardized protocols for administration, dosing, and treatment duration • Limited long-term data on efficacy and safety • Need for further validation through rigorous and comprehensive research • Potential for adverse effects, such as pain at the injection site, allergic reactions, and infection • Lack of insurance coverage, making it potentially costly or inaccessible for some patients • Operator-dependent: success heavily relies on the expertise and skills of the operator • Limited availability in healthcare settings and regions • Potential for ozone toxicity affecting the respiratory system • Lack of universal regulation; quality and safety standards may vary among practitioners and clinics

## Complications of ozone therapy

Ozone therapy, increasingly recognized in the scientific literature for its therapeutic potential, has demonstrated a commendable safety profile across various clinical applications when administered following established guidelines and employing an atoxic system. Clinical trials predominantly document mild and self-limiting adverse effects such as abdominal distension, lower limb hypoesthesia, and transient pain exacerbation, which typically resolve without necessitating extensive medical intervention [4, 21, 25, 26, 66]. Specifically, within the realm of musculoskeletal disorders, ozone therapy, in conditions like knee OA, has showcased a safety profile commensurate with hyaluronic acid injections, with both modalities yielding only mild, transient adverse events [33, 46]. This safety spectrum further extends to osteomyelitis, as evidenced in a rat model study, wherein ozone therapy augmented antioxidant mechanisms devoid of adverse reactions [14]. In treating chronic wounds and ulcers, studies have reiterated the minimal risk profile of ozone therapy, emphasizing its efficacy in wound healing [80]. Nonetheless, potential complications arising from specific administration methods, such as the use of polyvinyl chloride (PVC) auto-transfusion bags contaminated with excessive citrate–phosphate–dextrose (CPD), warrant vigilance [21]. Puncture accidents during therapy and risks in populations like the elderly or those with decompensated conditions merit careful consideration [10, 12].

Contraindications to ozone therapy are unambiguously defined, encompassing conditions like latent hypoglycaemia, hyperthyroidism, favism (due to G-6PD deficiency), pregnancy, and sickle cell anaemia. The use of angiotensin-converting enzyme (ACE) inhibitors also mandates caution with ozone therapy [20, 21]. These contraindications are rooted in potential risks such as the prospect of hemolysis in G-6PD deficiency, mutagenic concerns during early pregnancy, and asthmatic hypersensitivity.

Prolonged exposure to ozone may adversely affect the respiratory system, fostering the release of deleterious compounds into the bloodstream and potentially resulting in multiorgan damage [81]. Cells with deficient antioxidant activity are especially vulnerable to mutagenic alterations with sustained ozone exposure [82]. High concentrations of ozone can induce DNA oxidation and exhibit genotoxic effects [83]. Within the pulmonary milieu, ozone interaction with unsaturated fatty acids can yield lipid ozonation products, engendering lipid peroxidation, perturbed membrane permeability, and subsequent activation of inflammatory mediators [84]. When ozone commingles with nitrogen dioxide (NO_2_), the ensuing photochemical smog can intensify detrimental effects. Fortunately, antioxidants like vitamins E and C, along with anti-inflammatory agents like indomethacin, can serve as protective countermeasures against such adversities [20, 21].

## Limitations of ozone therapy

Ozone therapy, emerging as a promising therapeutic modality across diverse medical conditions, including osteonecrosis of the femoral head (ONFH) and orthopaedic disorders, has garnered considerable attention. Despite promising clinical observations [1, 5, 20, 28, 51, 69], the present study has methodological challenges which negatively impact the robustness of the findings. Notably, the prevalence of studies with small sample sizes underscores the imperative for investigations with expansive patient cohorts and prolonged follow-ups to affirm these preliminary insights [20, 46, 69, 80]. The methodological quality of much extant research tempers the interpretability of results. A significant portion of these studies exhibit a high or uncertain risk of bias [8, 12], highlighting the exigency for methodologically rigorous investigations to validate the therapeutic potential of ozone therapy [2]. A marked inconsistency in treatment protocols, as underscored by Fernandez-Cuadros et al. regarding knee OA [33], underlines the need for standardized approaches. Such consistency can lay the foundation for evidence-based guidelines, fostering the safe and efficacious deployment of ozone therapy in clinical contexts. A paramount lacuna in our current comprehension pertains to the elucidation of the exact biological and physiological mechanisms underpinning ozone therapy effects. This knowledge gap is accentuated in conditions like ONFH, where hypotheses around ozone role in neovascularization and immunoregulation remain speculative [1]. Sire et al. further highlight the necessity to broaden the research spectrum to encompass additional musculoskeletal disorders, enriching our understanding of ozone therapy applications in orthopaedics [18]. Deciphering these mechanisms is pivotal to refine and optimize treatment protocols. While the promise of ozone therapy in contexts such as wound healing in diabetic foot ulcers is evident [80, 85], the call for high-quality randomized controlled trials (RCTs) reverberates, especially when venturing beyond specific wound types. This is emblematic of the overarching need for comprehensive, methodologically sound trials which shed light on ozone therapy influence on inflammatory mediators and its broader clinical implications [3]. Despite ozone therapy prospective benefits, it encounters significant barriers to its mainstream medical integration, most prominently the absence of FDA approval and a paucity of evidence corroborating its expansive efficacy [17, 86, 87]. Overcoming these challenges demands the undertaking of well-constructed research studies, adhering to rigorous scientific paradigms. By redressing these research inadequacies, the scientific community can proffer compelling evidence which vindicates the place of ozone therapy in contemporary healthcare, emphasizing its efficacy and safety vis-à-vis conventional treatments [24, 75, 80].

## Level of evidence of ozone in orthopaedics

In this paper, the cited evidence is classified according to the Oxford Centre for Evidence-based Medicine hierarchy. Foremost, Level 2 evidence, encompassing randomized controlled trials (RCTs) and systematic reviews with meta-analyses, offers the most robust validation. This calibre of evidence prominently features in discussions on osteoarthritis, osteomyelitis, tendinopathies, rheumatic pathologies, and chronic pain syndromes. The methodological rigour of Level 2 studies engenders heightened confidence in the outcomes they present as mentioned in Table 3. Subsequently, Level 3 evidence, characterized by non-randomized controlled trials and case–control studies, emerges in contexts such as avascular necrosis, osteomyelitis, adhesive capsulitis, chronic pain syndromes, and fibromyalgia. Though not mirroring the exacting standards of Level 2 research, these Level 3 studies furnish indispensable foundational knowledge, potentially guiding subsequent, more rigorous investigations.

**Table 3:** Evidence levels for ozone interventions in various musculoskeletal disorders

Sl. no	Musculoskeletal disorder	Level of evidence
1	Fractures	IV
2	Osteoarthritis	II
3	Avascular necrosis	III
4	Osteomyelitis	III
5	Adhesive capsulitis	II
6	Tendinopathies	II
7	Rheumatic pathologies	II
8	Chronic pain syndromes	II
9	Fibromyalgia	II

Lastly, a solitary study, a systematic review assessing preclinical therapies for fracture non-union, is identified as Level 4 evidence. While systematic reviews are typically accorded a higher evidential standing, the specific methodological approach of this review positions it within the Level 4 category. In summary, the literature predominantly aligns with Level 2 and Level 3 evidence, reflecting a considerable degree of scientific rigour. The limited representation of Level 4 evidence, confined to a single systematic review of fractures, accentuates the depth of the findings while simultaneously highlighting domains warranting further high-level research to corroborate assertions across diverse medical contexts.

## Future prospective

The advancement of ozone therapy scientific foundation in orthopaedics demands stringent, meticulously designed randomized controlled trials with expanded sample sizes and prolonged follow-up periods [4, 8, 11, 15, 22]. Such studies should prioritize consistent, objective outcome measures to elevate their methodological quality, enabling a clearer comparison of various treatments [4]. It is essential that research impartially delineates the pros and cons of ozone therapy. A focal area of inquiry should be the interplay between constant oxidative stress and the episodic acute stress invoked by ozone treatments, a realm presently marked by uncertainty [40]. Deepening our comprehension of the molecular underpinnings guiding therapeutic responses is pivotal, not only to academic discourse but also in refining and personalizing treatment regimens for specific orthopaedic afflictions [1, 28, 65, 85]. For the therapy to achieve broader clinical acceptance, the establishment of standardized guidelines is paramount. These guidelines would encapsulate the best practices for ozone administration, thereby ensuring uniformity in therapeutic approaches and the consequent predictability of clinical outcomes [66]. The identification of differentially expressed genes between positive responders and non-responders to ozone therapy can offer insights into predicting therapeutic outcomes, facilitating a more personalized therapeutic strategy [1]. Research endeavours should encompass a detailed exploration of optimal dosages, session frequencies, and treatment durations, ensuring maximized therapeutic potential across varied orthopaedic conditions [3, 66]. Furthermore, expansive multicentre prospective studies can enhance the validation of ozone therapy’s therapeutic and safety profiles, extending its reach to broader patient demographics with a multitude of clinical conditions [1, 52, 88–92]. Emphasis should also be placed on examining the specific impacts of topical ozone treatments, such as ozonated water and oils, which currently lack comprehensive evaluation in human studies [75]. The fortification of ozone therapy’s role in orthopaedics necessitates a multifaceted research approach. This encompasses the execution of rigorous trials, a deeper investigation into molecular mechanisms, and the formulation of standardized treatment protocols. Through such integrated efforts, the orthopaedic community can establish a solid evidence base, ensuring the effective and reliable clinical integration of ozone therapy.

## Conclusion

Ozone therapy, with its multifaceted potential, is steadily gaining prominence across diverse clinical domains, notably within orthopaedics. At the physiological level, the therapy exhibits attributes crucial for orthopaedic interventions, including enhanced tissue oxygenation, modulation of oxidative stress, and anti-inflammatory properties. Such mechanisms provide potential relief in conditions such as knee OA, chronic osteomyelitis, and various chronic pain syndromes, positioning ozone therapy as a valuable adjunct or alternative when conventional treatments fall short. While the merits are evident, a comprehensive endorsement of ozone therapy in mainstream clinical practice awaits a more robust body of evidence. The imperative lies in rigorous research endeavours, particularly well-executed randomized controlled trials, to address current methodological limitations. Comprehensive studies must also delve into the precise mechanisms underpinning ozone therapy therapeutic impacts. Alongside this, the establishment of standardized treatment guidelines will be vital for its broader clinical acceptance and application. Until consolidated evidence emerges, clinicians should approach ozone therapy with circumspection, calibrating its use based on individual patient needs and clinical contexts. In essence, while ozone therapy offers considerable promise for a myriad of orthopaedic conditions, the onus is on the scientific community to further elucidate its efficacy, refine its application, and ascertain its safety profile.

## Data Availability

The datasets generated during and/or analysed during the current study are available throughout the manuscript.
